# Hepatitis B virus seromarkers among HIV infected adults on ART: An unmet need for HBV screening in eastern Ethiopia

**DOI:** 10.1371/journal.pone.0226922

**Published:** 2019-12-30

**Authors:** Desalegn Admassu Ayana, Andargachew Mulu, Adane Mihret, Berhanu Seyoum, Abraham Aseffa, Rawleigh Howe

**Affiliations:** 1 College of Health and Medical Sciences, Haramaya University, Oromia, Ethiopia; 2 Armauer Hansen Research Institute, Addis Ababa, Ethiopia; University of Cincinnati College of Medicine, UNITED STATES

## Abstract

Progression of chronic HBV to cirrhosis, end-stage liver disease (ESLD), and hepatocellular carcinoma (HCC) is more rapid in HIV positive individuals than those with HBV alone; however, the distribution of HBV seromarkers in HIV infected individuals on antiretroviral therapy (ART) is not well described. To address this problem, we assessed the distribution of hepatitis B surface antigen (HBsAg), hepatitis B core antibody (anti-HBc) and hepatitis B surface antibody (anti-HBs) among HIV infected adults on ART in Eastern Ethiopia. A cross-sectional study was conducted from September 2017 to February 2018. Socio-demographic, behavioral and health related factors, and clinical data were collected using questionnaire and checklist. Plasma samples were tested for HBsAg, anti-HBc and anti-HBs seromarkers using ELISA. Data were double entered into EpiData 3.1, cleaned, exported to and analyzed using STATA 13. Descriptive and logistic regression analysis were conducted and statistical significance was decided at p≤0.05. A total of 901 participants were included and the prevalence of HBsAg was found to be 11.7% [95%CI (10, 14)]. Among the co-infected, 47.6% were also positive for anti-HBc, of which 58% were on an ART containing tenofovir (TDF). Among those screened for the three seromarkers, 38.1% were negative for all and 21% were positive only for anti-HBc (IAHBc). Being single, history of genital discharge and taking ART with TDF combination were significantly associated with HBV co-infection (p≤0.05). There is high burden HBV co-infection among individuals on ART. The unmet need of HBV screening prior to ART initiation leaves many co-infected individuals without appropriate management including therapy, close monitoring or vaccination when indicated, impacting disease prevention.

## Introduction

Globally, 37.9 million (32.7 million-44.0 million) people were living with HIV in 2018, of which 20.6 million (18.2 million–23.2 million) were from the eastern and southern Africa region [1]. The proportion and risk factors of HBV co-infection vary widely with geographical location: 5–10% in North America, Europe and Australia, and 20–30% in sub-Saharan Africa (SSA) and Asia [2, 3].

HIV positive individuals are more likely to be infected with HBV than HIV-negative individuals, possibly as a result of shared risk factors [4]. Progression of chronic HBV to cirrhosis, end-stage liver disease (ESLD), and hepatocellular carcinoma (HCC) is more rapid in HIV co-infected individuals [5, 6].

HBV replication markers appear to be influenced by HIV infection [7]. HIV co-infection prevented HBsAg secretion with significantly elevated HBsAg quantity present in cell lysates in co-infected hepatic cell lines [8]. In HBV co-infection, HBsAg may hence be too low to be detected using serological tests and anti-HBc may be the only serological evidence of exposure to the virus [9]. In HBsAg negative individuals, anti-HBc positivity may be associated with occult hepatitis infection (OBI), characterized by low but detectable viral replication using PCR based approaches. [10]. HIV infection is also a risk factor for HBsAg negative HBV infection and the development of OBI, since it occurs more frequently in HIV-infected patients [11].

Testing and diagnosis of hepatitis infection is critical to both prevention and treatment services, and provides an opportunity to reduce transmission, through counselling on risk behaviors and vaccination [12]. HBV co-infection status is not commonly tested in ART clinics despite the recommendation for hepatitis B and C routine screening and vice versa [13]. Lack of HBV screening may lead to treatment without the use of the recommended tenofovir (TDF) in the regimen, which may be further associated with flares of hepatitis B due to ART-associated immune reconstitution [14]. In addition, the use of lamivudine (3TC) as the single agent in an ART regimen with activity against hepatitis B is contraindicated due to high likelihood of resistance development to YMDD (tyrosine-methionine-aspartate-aspartate), the highly conserved motif in HBV [15, 16].

In Ethiopia, reports indicate that HBsAg prevalence ranges from 2.7% [17] to 14% [18] among HIV infected individuals. Nearly all reports are based on HBsAg testing with less emphasis on anti-HBc and anti-HBs seromarkers despite their clinical importance. Screening for these seromarkers is also important to identify non-HBV exposed individuals who would benefit from HBV vaccination [13] or to identify those with chronic disease requiring follow-up monitoring or treatment.

This study therefore, assessed HBsAg, anti-HBc and anti-HBs seromarkers among HIV infected adults on ART to determine their magnitude, exposure related factors and reveal the unmet need of HBV screening.

## Materials and methods

### Study setting and period

The study was conducted in ART clinics of three selected public hospitals (Hiwot Fana Specialized University Hospital, Dilchora General Hospital, and Karamara General Hospital located in Harar, Dire Dawa and Jigjiga towns, respectively), in Eastern Ethiopia.

The hospitals provide specialist services to both the urban trade hubs and the pastoral and agro-pastoral communities inhabiting the borders with Somalia and Djibouti, each catering to an estimated five million populations. The study was conducted from September 2017 to February 2018.

### Study population

Consenting ART experienced HIV infected adults who attended ART clinic during the study period were recruited. The study included participants which were not HBV vaccinated and not screened for HBV seromarkers.

### Sample size determination

The sample size was calculated using OPEN Epi 3.1 assuming HBV co-infection of 6.9% [19] among HIV infected individuals on ART, and 3.7% among blood donors [20], and adding 10% for assumed non-response. The final calculated sample size was 920.

### Sampling procedure

A sample size of 385, 325 and 210 was allocated proportionally to Hiwot Fana, Dilchora and Karamara hospitals, respectively. Participants who were not HBV vaccinated and not previously screened for HBV were recruited consecutively at each site until the allocated sample size was attained.

### Data collection

Based on the routine follow-up of HIV infected individuals, an ART nurse screened eligible subjects, conducted interview and sent participants to the laboratory for sample collection with a unique identifier. History of opportunistic infection, history of tuberculosis (TB), baseline and current CD4+T cell count, duration on ART, initial and current ART regimen, and if changed, reason for regimen change, WHO clinical stages, medication adherence and other clinical and demographic information were captured from participants’ ART follow-up form using a checklist. A senior Internal Medicine specialist was involved for patient consultations as necessary at each site.

### Sample processing and serology

In the laboratory, 10 ml blood was collected in sterile anticoagulated tube, plasma was separated, labelled and stored at -20°C by medical technologists until transferred to Haramaya University. The plasma samples were screened and interpreted for HBsAg, ant-HBc and ant-HBs using ELISA (Monolisa HBsAg ULTRA, Monolisa Anti-HBc PLUS, Monolisa Anti-HBs PLUS, BIORAD, France) in the Medical Laboratory Science Department, College of Health and Medical Sciences, Haramaya University, following the manufacturer’s instruction.

### Quality control

To maintain data quality, data collectors were trained and questionnaire was pre-tested in different sites other than those selected for the study before the actual data collection. Samples were kept at -20°C until processed. Standard operating procedures (SOP) and pre-analytical, analytical and post analytical quality control measures were applied. ELISA test results were determined based on the cut-off values following the manufacturer’s instruction.

### Data management and analysis

Data were cleaned, coded and entered to EPI Data version 3.1 and analysed using Stata version 11(Stata Corp, USA). Descriptive analysis was used to calculate prevalence, and summarize sociodemographic and associated factors. A binomial logistic regression model was used and associated variables with p≤0.25 were entered to multiple regression analysis to control for potential confounders. Adjusted odds ratio (AOR) and 95% confidence interval (CI) were used to assess the strength of association between dependent and independent variables. Finally, statistical significance was decided at p<0.05.

### Ethical considerations

This study was reviewed and approved by the Institutional Health Research Ethics Review Committee (IHRERC) of the College of Health and Medical Sciences, Haramaya University (Ref. No. IHRERC/137/2017) and AHRI/ALERT Ethics Review Committee, Addis Ababa (Ref. No. P019/17). Written signed informed consent was obtained before data collection. Laboratory results of HBV seromarkers were reported to the respective attending clinician for the necessary intervention. To maintain confidentiality, participants’ information was coded and names and personal identifiers were not used.

## Results

### Socio-demographic characteristics of study participants

A total of 901 (98%) HIV infected individuals on ART were included in this study. Of the total participants, 817 (90.7%) were urban residents, and 622 (69%) were female of which 539 (86.6%) were within the reproductive age group. The median age of the respondents was 40 years (IQR 32, 45) with an average family size of 3.12. Among the study participants, 291 (32.3%) did not attend formal education, and 71 (7.9%), had attended tertiary education. “Table 1”

**Table 1:** Socio-demographic characteristics of HBV co-infected and HIV mono-infected individuals on ART in selected public hospitals, Eastern Ethiopia, 2017/18 (n = 901).

Characteristics		HBsAg	
		Positive n (%)	Negative n (%)
Sex	Female	74 (70.5)	548 (68.8)
	Male	31 (29.5)	248 (31.2)
Age (Years)	15–21	6 (5.7)	40 (5.0)
	22–34	33 (31.4)	190 (23.9)
	35–49	52 (49.5)	428 (53.8)
	≥50	14 (13.3)	138 (17.3)
Residence	Urban	92 (87.6)	725 (91.1)
	Rural	13 (12.4)	71 (8.9)
Family size	1–5	92 (87.6)	728 (91.5)
	≥6	13 (12.4)	68 (8.5)
Marital status	Single	30 (28.6)	172 (21.6)
	Married	34 (32.4)	346 (43.5)
	Divorced	41 (39.0)	278 (34.9)
Level of education	No formal education	32 (30.5)	259 (32.5)
	Primary and secondary	31 (29.5)	303 (38.1)
	High school	31 (29.5)	174 (21.9)
	College/university	11 (10.5)	60 (7.5)
Occupation	Government employee	18 (17.1)	128 (16.1)
	Student	7 (6.7)	40 (5.0)
	Merchant	5 (4.8)	84 (10.6)
	House wife	23 (21.9)	146 (18.3)
	Private/self-employee	9 (8.6)	105 (13.2)
	Unemployed	19 (18.1)	135 (17.0)
	Daily labourer	17 (16.2)	111 (13.9)
	Others	7 (6.7)	47 (5.9)

### Behavioral and health related characteristics of study participants

Of the screened HIV infected individuals on ART, 173 (19.2%) reported current alcohol consumption, 118 (13.1%) khat chewing, 544 (60.4%) a history of body piercing, 293 (32.5%) had tattoos and 314 (34.9%) shared sharp tools such as needles and razors. Three hundred sixty-two (40.2%) had history of hospital admission, 59 (6.5%) genital discharge, 186 (20.6%) history of dental extraction, and 119 (13.8%) had multiple sexual contacts.

### Distribution of HBsAg, anti-HBc and anti-HBs seromarkers among study participants

Based on the three HBV seromarkers assessed, 334 (38.1%) were negative for all, 176 (20.1%) were positive for both anti-HBc and anti-HBs and 53 (6.0%) were positive only for HBsAg. Those positive for “anti-HBc only” (IAHBc) totaled 184 (21.0%), of which 113 (61.4%) were females and 93% of these females were urban residents. “Table 2”

**Table 2:** Distribution of HBV seromarkers among HIV infected individuals on ART in selected public Hospitals, Eastern Ethiopia, 2017/18 (n = 876).

HBV seromarkers	Results	Interpretation	Frequency
			n (%)
HBsAg	Negative	Susceptible/no evidence of prior infection	334 (38.13)
Anti-HBc	Negative		
Anti-HBs	Negative		
HBsAg	Negative	Immune due to natural infection	176 (20.09)
Anti-HBc	Positive		
Anti-HBs	Positive		
HBsAg	Positive	Chronically infected based on Anti HBc total	42 (4.79)
Anti-HBc	Positive		
Anti-HBs	Negative		
HBsAg	Positive	*Unclear	53 (6.05%)
Anti-HBc	Negative	1. Early infection/low Anti-HBc level	
Anti-HBs	Negative	2. False positive HBsAg, thus susceptible	
HBsAg	Negative	Unclear	184 (21.0)
Anti-HBc	Positive	1. Resolved infection (most common)	
Anti-HBs	Negative	2. False positive Anti HBc, thus susceptible	
		3. “Low level” chronic infection	
		4. Resolving acute infection	
		**5. Isolated anti-HBc (IAHBc)	

* HBsAg only positive was not included in the advisory committee recommendations

** not included in the advisory committee recommendations

Adapted from CDC, Recommendations of the Advisory Committee on Immunization Practices (ACIP) 2006.

### Clinical characteristics of HBV co-infected and HIV mono-infected individuals

Nearly all, 99.8%, of the study participants were on ART, and had been receiving treatment for a median duration of 86.0 months (IQR 51,118). A total of 850 (95.6%) were taking first line and 39 (4.4%) were taking second line ART regimen currently. From a total of 313 (35.2%) who had ART regimen changed, 144 (46.0%) were due to undesirable side effects and 39 (12.5%) were due to apparent treatment failure. Among participants taking second line drugs due to treatment failure, 3 (7.5%) were HBsAg positive, and one was in WHO clinical stage III category.

Based on the clinical data record of the study participants, 244 (27.2%) had history of tuberculosis (TB) and 476 (53.1%) had a history of opportunistic infections (OI). The vast majority of the participants, 835 (93.8%) had good ART adherence, and 844 (94.8%) were in WHO stage I category. “Table 3”

**Table 3:** Clinical characteristics of HBV co-infected and HIV mono-infected individuals on ART in selected public hospitals, Eastern Ethiopia, 2017/18.

Characteristics		HBsAg	
		Positive n (%)	Negative n (%)
History of TB (n = 897)	Yes	31 (29.5)	213 (26.9)
	No	74 (70.5)	579 (73.1)
History of OI (n = 897)	Yes	81 (77.1)	395 (49.9)
	No	24 (22.9)	397 (50.1)
Baseline CD4 T cells/mm^3^ (n = 829)	≤200	57 (57.6)	424 (58.1)
	201–350	32 (32.3)	192 (26.3)
	351–500	5 (5.1)	50 (6.8)
	≥501	5 (5.1)	64 (8.8)
Current CD4 T cells/mm^3^ (n = 798)	≤200	7 (7.1)	56 (8.0)
	201–350	16 (16.3)	117 (16.7)
	351–500	19 (19.4)	163 (23.3)
	≥501	56 (57.1)	364 (52.0)
Current ART with TDF and 3TC (n = 889)	Yes	57 (54.3)	510 (65.1)
	No	48 (45.7)	274 (34.9)
Current ART regimen (n = 889)	First line	102 (97.1)	748 (95.4)
	Second line	3 (2.9)	36 (4.6)
Regimen change (n = 889)	Yes	40 (38.1)	273 (34.8)
	No	65 (61.9)	511 (65.2)
Reason for regimen change (n = 313)	New drug	11 (27.5)	78 (28.6)
	Side effect	24 (60.0)	120 (44.0)
	Treatment failure	3 (7.5)	36 (13.2)
	Others	2 (5.0)	39 (14.3)
Duration on ART (n = 889)	≤6 months	2 (1.9)	31 (4.0)
	>6 months	103 (98.1)	753 (96.0)
WHO clinical stage (n = 890)	I	101 (96.2)	743 (94.6)
	II	3 (2.9)	27 (3.4)
	III	1 (1.0)	13 (1.7)
	IV	0	2 (0.3)
ART adherence (n = 890)	Good	101 (96.2)	734 (93.5)
	Fair	3 (2.9)	37 (4.7)
	Poor	1 (1.0)	14 (1.8)

**NB**: The total number varies based on the complete record available for each variable.

*Adherence was assessed using percent of adhered days and % missed doses.

The median baseline and current CD4+T cells counts of the participants were 180 and 519 cells/μl (IQR: 104.5, 274.5; 353.75, 691), respectively. Among the total participants, 481 (58.0%) had baseline CD4+ T cells count ≤200 cells/μL. Among these, 57 (11.9%) were HBsAg positive, of which 23 (40.4%) were taking ART combination with TDF and 3TC. Currently, 63 (7.9%) had CD4+ T cells count ≤200 cells/μL.

From HBV co-infected individuals, 31 (29.5%) had a history of tuberculosis, 81 (77.1%) had history of opportunistic infections and 101 (96.2%) had good adherence and were in the WHO clinical stage I category.

A total of 567 (63.8%) participants were currently taking ART containing TDF and 3TC combinations. From those who were both HBsAg and anti-HBc positive, 29 (58%) were currently taking an ART containing both TDF and 3TC, however, 21 (42%) were taking ART regimens comprising 3TC only.

### Prevalence of Hepatitis B virus surface antigen and associated factors

Among the study participants, 105 [11.7%, 95%CI (10–14)] were positive for HBsAg. The distribution was 11.9% among females and 11.1% among males. The majority, 92 (87.6%), of the HBsAg positives were urban residents and 74 (70.5%) were female. Nearly half, 50 (47.6%) of the HBsAg positives were also positive for anti-HBc, among which, 42 (84%) were urban residents, and 31 (62.0%) were females, of which 27 (87.1%) were within the reproductive age group.

Binary logistic regression analysis was conducted including sociodemographic, behavioral, health related and clinical variables. Marital status, tattooing, hospital admission, sharing sharp tools, discharge from genitalia, genital mutilation, history of opportunistic infections and presence of TDF and 3TC in the ART regimen were considered for multivariable logistic regression analysis (p≤0.25). In the final model, marital status, history of genital discharge and ART with TDF and 3TC combination remained statistically significant (p<0.05). Besides, sharing sharp tools and genital mutilation remained strong predictors though they were marginally significant.

HIV infected individuals who were single were 2 times more likely to be HBsAg positive compared to married ones [AOR 2.10; 95%CI (1.03, 4.28) p = 0.041]. History of genital discharge increased the chance of being HBsAg positive by 2.9 times [AOR 2.90; 95%CI (1.18, 7.09) p = 0.020]. Those who were currently treated with ART without TDF were 1.9 times more likely to be HBsAg positive compared to those treated with TDF combinations [AOR 1.89; 95%CI (1.10, 3.23) p = 0.020]. Likewise, those who shared sharp tools were 1.9 times more likely and females who had genital mutilation were 1.8 times more likely to be HBsAg positive compared to their counterparts. However, the p value was marginally significant [AOR 1.97; 95%CI (0.99, 3.93) p = 0.051; AOR 1.81; 95%CI (0.99, 3.28) p = 0.052]. “Table 4”

**Table 4:** Significant predictors of HBsAg positivity among HIV infected individuals on ART in selected public hospitals, Eastern Ethiopia, 2017/2018 (n = 901).

Characteristics		HBsAg		COR (95%CI	AOR (95%CI	P
		Positive n (%)	Negative n (%)			
Marital status	Single	30 (28.6)	172 (21.6)	1.77 (1.05–2.99)	2.10 (1.03–4.28)	0.041*
	Married	34 (32.4)	346 (43.5)	1	1	0.770
	Divorced	41 (39.0)	278 (34.9)	1.50 (0.93–2.43)	1.09 (0.58–2.05)	
Tattooing	Yes	50 (47.6)	243 (30.5)	2.06 (1.37–3.12)	1.61 (0.89–2.91)	0.112
	No	55 (52.4)	553 (69.5)	1	1	
Hospital admission	Yes	55 (52.4)	307 (38.6)	1.75 (1.16–2.64)	1.20 (0.70–2.07)	0.502
	No	50 (47.6)	489 (61.4)	1	1	
Share sharp tools	Yes	63 (60.0)	251 (31.5)	3.26 (2.14–4.95)	1.98 (0.99–3.93)	0.051
	No	42 (40.0)	545 (68.5)	1	1	
Genital discharge	Yes	12 (11.4)	47 (5.9)	2.05 (1.05–4.02)	2.90 (1.18–7.09)	0.020*
	No	93 (88.6)	749 (94.1)	1	1	
Genital mutilation (n = 622)	Yes	51 (68.9)	240 (43.8)	2.84 (1.69–4.79)	1.81 (0.99–3.28)	0.052
	No	23 (31.1)	308 (56.2)	1	1	
History of OI (n = 897)	Yes	81 (77.1)	395 (49.9)	3.39 (2.11–5.46)	1.36 (0.64–2.87)	0.421
	No	24 (22.9)	397 (50.1)	1	1	
Baseline CD4 T cells/mm^3^ (n = 829)	≥500	5 (5.1)	66 (9.0)	1	1	
	<499	94 (94.9)	664 (91.0)	1.87 (0.73–4.75)	5.79 (0.77–43.67)	0.088
Current ART with TDF and 3TC (n = 889)	Yes	57 (54.3)	510 (65.1)	1	1	
	No	48 (45.7)	274 (34.9)	1.56 (1.04–2.36)	1.88 (1.10–3.23)	0.020*

COR = crude odds ratio AOR = Adjusted odds ratio

*Statistically significant variables

### Prevalence of Anti-HBc and associated factors among study participants

A total of 419 [(46.5%, 95%CI (43, 50)] HIV infected individuals on ART were positive for anti-HBc seromarkers, among which the majority, 272 (64.9%) were female and 383 (91.4%) were urban residents. The distribution was 43.7% among female and 52.7% among male.

To assess independent predictors of anti-HBc positivity, factors that were significant in bivariate analysis (p≤0.25) were entered into multivariable logistic regression analysis. In the final model gender, level of education, occupation and multiple sexual contact remained significant predictors (p≤0.05). Males were 1.6 times more likely to be anti-HBc positive [AOR; 1.59 95%CI (1.11, 2.26) p = 0.010]. Unemployed participants were 2 times and daily laborers were 1.9 times more likely to be anti-HBc positive compared to government employees [AOR; 2.17 95%CI (1.28, 3.67) p = 0.004; AOR; 1.90 95%CI (1.11, 3.26) p = 0.020]. Those who had multiple sexual contacts were 2 times more likely to be anti-HBc positive compared to their counterparts [AOR; 2.21 95%CI (1.44, 3.38) p = 0.001]. High school students were 63% less likely of being anti-HBc positive compared to college or university graduates [AOR; 0.37 95%CI (0.19, 0.69) p = 0.002]. “Table 5”

**Table 5:** Significant predictors of anti-HBc positive among HIV infected individuals on ART in selected public hospitals, Eastern Ethiopia, 2017/2018.

Characteristics		Anti-HBc		COR (95%CI)	AOR (95%CI)	P
		Positive n (%)	Negative n (%)			
Gender	Female	272 (64.9)	350 (72.6)	1	1	
	Male	147 (35.1)	132 (27.4)	1.43 (1.08–1.90)	1.65 (1.16–2.35)	0.005*
Age	15–21	5 (1.2)	41 (8.5)	1	1	
	22–34	88 (21.0)	135 (28.0)	5.34 (2.03–14.05)	1.13 (0.18–7.23)	0.894
	35–49	249 (59.4)	231 (47.9)	8.84 (3.43–22.75)	1.73 (0.27–10.96)	0.560
	> = 50	77 (18.4)	75 (15.6)	8.42 (3.15–22.46)	1.47 (0.22–9.64)	0.685
Marital status	Single	69 (16.5)	133 (27.6)	0.55 (0.39–0.78)	0.71 (0.46–1.07)	0.108
	Married	184 (43.9)	196 (40.7)	1	1	
	Divorced	166 (39.6)	153 (31.7)	1.15 (0.85–1.55)	1.11 (0.78–1.56)	0.552
Level of Education	No formal education	141 (33.7)	150 (31.1)	0.86 (0.51–1.45)	0.56 (0.30–1.04)	0.070
	Primary and secondary	166 (39.6)	168 (34.9)	0.91 (0.54–1.51)	0.61 (0.33–1.12)	0.111
	High school	75 (17.9)	130 (27.0)	0.53 (0.31–0.91)	0.38 (0.20–0.71)	0.002*
	College/University	37 (8.8)	34 (7.1)	1	1	
Occupation	Government employee	64 (15.3)	82 (17.0)	1	1	
	Private/Self-employee	60 (14.3)	54 (11.2)	1.42 (0.87–2.32)	1.58 (0.92–2.73)	0.095
	Student	6 (1.4)	41 (8.5)	0.18 (0.07–0.47)	0.31 (0.03–3.15)	0.324
	Merchant	42 (10.0)	47 (9.8)	1.14 (0.67–1.94)	1.33 (0.75–2.36)	0.325
	House wife	73 (17.4)	96 (19.9)	0.97 (0.62–1.52)	1.31 (0.77–2.22)	0.318
	Unemployed	84 (20.0)	70 (14.5)	1.54 (0.97–2.42)	2.07 (1.23–3.48)	0.006*
	Daily laborer	69 (16.5)	59 (12.2)	1.49 (0.93–2.41)	1.82 (1.06–3.11)	0.029*
	Others	21 (5.0)	33 (6.8)	0.81 (0.43–1.54)	0.82 (0.41–1.64)	0.574
Multiple sexual contacts	Yes	75 (18.2)	44 (9.8)	2.04 (1.37–3.04)	2.22 (1.45–3.39)	0.001*
	No	338 (81.8)	405 (90.2)	1		
Dental extraction	Yes	94 (22.4)	92 (19.1)	1.22 (0.89–1.69)	099 (0.71–1.41)	0.999
	No	325 (77.6)	390 (80.9)	1		
Genital discharge	Yes	35 (8.4)	24 (5.0)	1.74 (1.01–2.97)	1.53 (0.88–2.68)	0.133
	No	384 (91.6)	458 (95.0)	1		

## Discussion

The overall prevalence of HBsAg and anti-HBc among HIV infected individuals on ART was 11.7% and 46.5%, respectively. Among the HBsAg positives, 47.6% were also positive for anti-HBc and (29/50) 58% of these were currently on an ART regimen containing TDF and 3TC. Among the total participants screened for the three seromarkers, 38.1% were negative for all, and 21% were positive only for anti-HBc (IAHBc), and 96% of the participants were on ART for more than 6 months. Being single, history of genital discharge and taking ART with TDF were significant predictors of HBV co-infection. Male gender, unemployment, daily laborer, history of multiple sexual contacts and level of education were significant predictors of HBV exposure.

The HBV co-infection prevalence in this study is higher compared to HBV prevalence of 4.7% in Addis Ababa [21], 5.9% in Mekelle hospital [22], 5.5% in University of Gondar Hospital [23], 6.3% in Southern Ethiopia [24], 6.9% in Hawassa Referral Hospital [25], a pooled prevalence of 5.2% in meta-analysis regardless of ART status [26] and a 7.4% global prevalence report [27]. However, it is less than the 14% HBV prevalence reported from Shashemene town in Southern Ethiopia [18] and the overall prevalence of 15% in sub-Saharan Africa [28]. A similar finding of 11.7% was reported among HIV infected HAART naïve individuals in north-west Gondar [29]. A recent population-based HIV impact assessment (EPHIA 2017–2018) report in Ethiopia indicated a 4.8% HBV co-infection among adults of 15–64 years of age (3.6% in women to 7.4% in men) in urban Ethiopia using rapid diagnostic test [30]. The variations in prevalence reports may be attributed to ART exposure that could significantly reduce the level of HBV DNA [31], geographical variation of HBsAg carriage that range from 1.9% to over 40% [32] and mutations in the S region of HBV [6]. The difference in sample size and the diagnostic tools may also affect to the prevalence reports in Ethiopia.

The high HIV/HBV co-infection in this report is an evidence for the serious health burden that demands immediate intervention considering the recent HIV increase from 1.14% in 2014 [15] to 3% in 2018 in urban population in Ethiopia [30] due to the shared risk factors. Additionally, 87.1% of those females who were positive for both HBsAg and anti-HBc were within the reproductive age group. This increases the risk of vertical and horizontal transmission, as well as progression to chronic liver disease unless the necessary precautions are implemented.

Studies reported that one-third of the world’s population has serologic evidence of past or present HBV infection [33]. Though anti-HBc is widely used in HBV screening as an epidemiological marker [34], data on the magnitude of anti-HBc is limited in Ethiopia. The anti-HBc seropositivity in this study is higher than the 22.5% anti-HBc prevalence reported in southern Ethiopia [24], and slightly lower compared to the 52.4% reported among ART experienced patients in Addis Ababa [35] and the 55.1% reported from sub-Saharan Africa [28]. About 70–90% of all HIV patients show evidence of past or active HBV infection in Kenya [32]. The possible explanation for such variations could be the simultaneous infection with HIV that reduces immune control of previous HBV infection facilitating HBV reactivation and HBV DNA replication without presence of detectable HBsAg [36]. Moreover, immune status of the study participants and stages of HBV disease [37] may affect likelihood of HBsAg detection. The higher occurrence of occult HBV infection in HIV-positive people may also be attributed to the lower rate of HBsAg [4]. The anti-HBc carriage among HIV infected individuals on ART may indicate a high rate of HBV transmission as anti-HBc typically persists for life, regardless of whether the infection resolves or remains chronic [38]. However, the anti-HBc test should be supported by testing for HBsAg and anti-HBs in order to decide whether it indicates HBV immunity through natural infection, chronic HBV infection, IAHBc [39] or OBI [40].

In our study, based on the seromarkers assessed, 37.3% of the study participants were negative for HBsAg, anti-HBc and anti-HBs, and therefore susceptible to HBV infection [39]. These group could be protected from HBV infection through vaccination if pre-ART HBV screening had been implemented in Ethiopia. The unmet need of pre-ART HBV screening and subsequent appropriate vaccination hampered the HBV infection control effort [13], and as well as contributing to progression of untreated chronic HBV to cirrhosis, ESLD, and HCC [5, 6]. Similarly, 20.1% were positive for both anti-HBc and anti-HBs indicating protection due to natural infection, and 4.8% were positive for both HBsAg and anti-HBc indicating the requirement for further follow up, to prevent adverse consequences [39]. Though it was not defined in the advisory committee recommendations[39], we found 6.1% of HBsAg only positive cases. This might have occurred due to low anti-HBc levels depending on the immune tolerant and inactive phases of HBV infection which may affect its detection [41].

Furthermore, 21% of the total screened were positive for anti-HBc and negative for both HBsAg and anti-HBs, hence categorized as “isolated anti-HBc” (IAHBc), or “anti-HBc alone” [42]. In Ethiopia, we have not observed any data reporting IAHBc to date and therefore, the burden is unknown despite its clinical and public health implications. IAHBc may represent several clinical entities including the window phase of acute HBV when anti-HBs is not yet detected, the late stage of prior infection after anti-HBs has fallen to undetectable levels, OBI, or false positive anti-HBc [40, 43]. HIV co-infection was demonstrated to be a risk factor for HBsAg negative infection [11, 44], and pre-S1 [45] and ‘a’ determinant mutations also prevent HBsAg secretion, ultimately affecting virus detection [46, 47]. High anti-HBc levels in the immune active and immune reactivation phases of chronic HBV infection [41] and the higher occurrence of OBI (2% to 10%) in HIV-positive people may also affect detection rate of HBsAg [48, 49], leading to IAHBc. Patients who have undergone viral clearance may also lose the ability to produce anti-HBs after long periods of time due to a waning T-cell response [40]. OBI has much impact on different clinical and public health aspects, including transmission, risk of reactivation and enhancing liver disease progression that can lead to HCC [50]. This further emphasizes the critical need of HBV screening among HIV infected individuals before ART initiation to prevent the potential risk of HBV transmission.

In our study, nearly all study participants were on ART with different combinations for a median of 86 months. For HBV co-infected individuals if treatment is indicated, the national guideline recommends TDF + 3TC (or FTC) + EFV as a preferred regimen [51]. We found that 70% of the participants had been treated with an ART regimen containing TDF compared to a the 51.2% report among HBsAg and anti-HBc positive individuals on ART [52]. The rest were on ART with 3TC as the only anti-HBV-active agent. This may cause unnecessary consequences on the patients due to continued HBV viremia and progression [53], resulting from high rates of drug resistance mutations (DRMs) and virologic breakthrough [54]. Furthermore, 3TC resistance confers partial or complete cross-resistance to other HBV inhibitors such as emtricitabine (FTC), telbivudine and entecavir (ETV), thus limiting treatment options [15]. In Ethiopia, the unmet need of HBV screening, might have affected the clinical outcome of the patients who were eligible for TDF but remain taking 3TC only leading to circulation of drug resistant strains among the community.

Being single, history of genital discharge, and ART without TDF [31] were statistically significant predictors of HBsAg positivity. This study, did not find a statistically significant association between HBsAg positivity and many of the sociodemographic characteristics such age, sex, residence and level of education like other similar studies [18, 21, 22, 24, 55]. History of hospital admission, surgery, dental extraction [22, 55] and sharing of sharp materials [25, 55] were not significantly associated with HBsAg.

Male gender [35], occupation (being unemployed and daily laborer), level of education and multiple sexual partner were significant predictors for HBV exposure. The risk difference by gender may be due to differences in risk behavior over years of life [56]. However, exposure to HBV infection was not significantly associated with body piercing and tattooing [24], and age and history of genital discharge. No significance difference was observed by residence, marital status and history of dental extraction as a risk for HBV exposure [24, 35]. Those who shared sharp tools were 1.9 times more likely and females who had genital mutilation were 1.8 times more likely to be HBsAg positive compared to their counterparts. However, the p value remained statistically insignificant (p>0.05).

### Limitations

This study did not conduct HBeAg and anti-HBc IgM tests to differentiate between active viral replication and acute infection, respectively among the study participants. HIV viral load, liver function tests and related data were incomplete to assess other important clinical parameters.

## Conclusion and recommendations

This study showed a relatively high prevalence of HIV/HBV coinfection, HBV co-infected participants taking 3TC as the only anti-HBV agent, susceptible HIV infected adults that require HBV vaccine and IAHBc group. In general, the unmet need for HBV screening prior to ART initiation may affect the quality of care given to the patients, increase the risk of life-threatening complications among the co-infected, but untreated ones and hamper the prevention effort towards HIV and HBV as they share transmission routes. In addition, it brings into attention the importance of integrating anti-HBc screening due to its public health implication in HBV transmission as well as drug resistant mutations.
